# Revisiting the standard of care for immune checkpoint inhibitors in early-stage triple-negative breast cancer: timing, duration, dose, combination, and patient selection

**DOI:** 10.3389/fimmu.2026.1795426

**Published:** 2026-04-17

**Authors:** Xi Guo, Panni Li, Wei Wu, Yanyan Wang, Jie Qiu, Qianyi Ma, Xianan Guo, Kexin Liu, Yali Wang, Wenhui Ruan, Sifeng Tao, Zhengyang Xu, Yulan Shi, Yunxiang Zhou

**Affiliations:** 1 Nursing Department, The Second Affiliated Hospital of Zhejiang University School of Medicine, Hangzhou, Zhejiang, China; 2 Department of Breast Surgery and Oncology, The Second Affiliated Hospital, Zhejiang University School of Medicine, Hangzhou, Zhejiang, China; 3 Cancer Institute (Key Laboratory of Cancer Prevention and Intervention, China National Ministry of Education), The Second Affiliated Hospital, Zhejiang University School of Medicine, Hangzhou, China; 4 Department of Medical Oncology, Zhejiang Cancer Hospital, Hangzhou Institute of Medicine (HIM), Chinese Academy of Sciences, Hangzhou, Zhejiang, China; 5 Department of Breast and Thyroid Surgery, Affiliated Shaoxing Hospital, Zhejiang University School of Medicine, Shaoxing, China; 6 Department of Pharmacy, Second Affiliated Hospital, Zhejiang University School of Medicine, Hangzhou, China; 7 Department of Breast Surgery, Fujian Medical University Union Hospital, Fuzhou, Fujian, China; 8 Breast Cancer Institute, Fujian Medical University, Fuzhou, Fujian, China; 9 Department of Thyroid and Breast Surgery, People’s Hospital of Anji, Huzhou, Zhejiang, China; 10 Department of Thyroid and Breast Surgery, The First Affiliated Hospital, Zhejiang University School of Medicine, Hangzhou, Zhejiang, China; 11 Department of Tumor Radiotherapy and Chemotherapy, The Affiliated People’s Hospital of Ningbo University, Ningbo, Zhejiang, China

**Keywords:** triple-negative breast cancer, immune checkpoint inhibitor, neoadjuvant immunotherapy, adjuvant immunotherapy, KEYNOTE-522, standard of care, treatment optimization, treatment de-escalation

## Abstract

Triple-negative breast cancer (TNBC) is a highly immunogenic breast cancer subtype, rendering it particularly amenable to immunotherapy. Immune checkpoint inhibitors (ICIs), primarily targeting the PD-1/PD-L1 axis, have transformed the therapeutic landscape of early-stage TNBC, with KEYNOTE-522 trial establishing neoadjuvant pembrolizumab (continued as adjuvant) plus chemotherapy as the current standard of care. However, the substantial toxicity associated with the KEYNOTE-522 regimen, together with residual uncertainty regarding the relative contributions of neoadjuvant versus adjuvant pembrolizumab administration, underscores the need to optimize treatment intensity and refine ICI strategies. Herein, we provide an overview of immunotherapy and its clinical applications in TNBC. We integrate evidence from neoadjuvant and adjuvant ICI trials with mechanistic insights into the biologically optimal timing of immunotherapy. We further highlight emerging strategies aimed at optimizing the current standard-of-care regimen, with the potential to refine treatment timing, duration, dose, combination strategies, and patient selection for ICI therapy, thereby providing insights into future therapeutic approaches for early-stage TNBC.

## 1 Introduction

Breast cancer is the most common malignancy worldwide and a leading cause of cancer-related death (1). Triple-negative breast cancer (TNBC), characterized by the absence of estrogen receptor, progesterone receptor, and HER2 overexpression, accounts for approximately 15–20% of all breast cancer cases yet contributes nearly 40% of breast cancer–related deaths (2). Compared with hormone receptor (HR)-positive and HER2-positive subtypes, TNBC displays more aggressive biological behavior, with earlier and more frequent recurrence in early-stage disease, as more than half of patients relapse within 3–5 years of diagnosis. Survival also declines substantially in the advanced setting, where the median overall survival (OS) remains approximately 10.2 months (3, 4).

Immune checkpoint inhibitors (ICIs), a major class of immunotherapy, have demonstrated clinical efficacy in advanced TNBC and, notably, in early-stage disease. The KEYNOTE-522 trial (5–7) showed that pembrolizumab combined with neoadjuvant chemotherapy (NACT), followed by adjuvant pembrolizumab, improved pathological complete response (pCR) (5) and survival (6, 7), establishing the standard of care (SoC) for high-risk early-stage TNBC.

However, the KEYNOTE-522 regimen is associated with substantial toxicity (5), raising questions about the generalizability of the current SoC. In KEYNOTE-522, pembrolizumab was given both before and after surgery, leaving it unclear whether the benefit derived primarily from the neoadjuvant, adjuvant, or combined phases. Several studies have also evaluated ICIs exclusively in the adjuvant setting. The recent A-BRAVE trial in high-risk early-stage TNBC patients showed that one year of adjuvant avelumab did not improve 3-year disease-free survival (DFS), yet unexpectedly appeared to confer a benefit in 3-year OS (8). These findings have reignited discussion on revisiting the SoC in early-stage TNBC, particularly regarding the timing of immunotherapy and opportunities for treatment optimization.

In this review, we first provide an overview of immunotherapy, including its definition, major categories, mechanisms of action, safety profile, and current clinical applications in TNBC. We then summarize the evidence for ICI use in both the neoadjuvant and adjuvant settings, and discuss mechanistic insights that may inform the biologically optimal timing of immunotherapy. Finally, we outline emerging strategies aimed at refining and optimizing the current SoC for perioperative ICI-based therapy, with the goal of guiding future directions in the management of early-stage TNBC.

## 2 Immunotherapy and its mechanisms of action

Immunotherapy aims to enhance the ability of the immune system to recognize and eliminate tumor cells, thereby reshaping antitumor immunity within the tumor microenvironment (TME) (9). Among the established therapeutic approaches, ICIs represent the most prevalent and clinically validated class. These agents block key immune checkpoint molecules, including programmed cell death protein 1 (PD-1), its ligand programmed death-ligand 1 (PD-L1), and cytotoxic T-lymphocyte-associated protein 4 (CTLA-4), either alone or in combination, and constitute the foundation of current immunotherapeutic strategies (9). In addition to the conventional immune checkpoint pathways, other immune checkpoint proteins have been identified, including lymphocyte activation gene-3 (LAG-3), T cell immunoreceptor with Ig and ITIM domains (TIGIT), mucin domain–containing protein 3 (TIM-3), B and T lymphocyte attenuator (BTLA), V-domain immunoglobulin suppressor of T cell activation (VISTA), and signal-regulatory protein α (SIRPα) (10). To date, the U.S. Food and Drug Administration (FDA) has approved PD-1 inhibitors (pembrolizumab, nivolumab, cemiplimab, dostarlimab), PD-L1 inhibitors (durvalumab, atezolizumab, avelumab), CTLA-4 inhibitors (ipilimumab, tremelimumab), and the LAG-3 inhibitor relatlimab (11). Beyond ICIs, several additional immunotherapeutic strategies have been developed, including chimeric antigen receptor (CAR) T-cell therapy and bispecific T-cell engagers (BTEs). Other investigational approaches involve stimulator of interferon genes (STING) agonists, targeted cytokine delivery, and methods designed to enhance neoantigen recognition, such as vaccines, oncolytic viral therapies, and radiation-induced immunogenicity (9).

Under physiological conditions, immune checkpoint molecules such as PD-1 and PD-L1 function to maintain immune homeostasis and prevent excessive immune activation (12, 13). Tumor cells, however, can exploit these pathways as an adaptive immune evasion strategy, most notably through upregulation of PD-L1 to engage PD-1 on activated T cells. This interaction suppresses antitumor immune responses and allows tumor cells to evade immune-mediated destruction (12). By blocking immune checkpoint signaling, ICIs restore T-cell effector function, enabling immune cells to recognize and eliminate tumor cells that were previously tolerated or undetected by the immune system (14). This immune reactivation, while central to the antitumor efficacy of ICIs, also underlies the development of immune-related adverse events (irAEs), as it can provoke off-target immune activation against normal tissues, resulting in autoimmune toxicities (15, 16).

Combination strategies integrating immunotherapy with other systemic anticancer treatments, such as cytotoxic chemotherapy and antibody–drug conjugates (ADCs), can exert complementary and synergistic immunomodulatory effects (10, 17). Chemotherapeutic agents (e.g., anthracyclines, taxanes, and cyclophosphamide) are capable of inducing immunogenic tumor cell death, leading to the release of tumor-associated antigens and danger signals that enhance antigen presentation, dendritic cell maturation, and T-cell priming (18). In this context, chemotherapy not only reduces tumor burden but also modulates the TME toward a more immunogenic state, thereby facilitating a more effective immune response to subsequent or concurrent immunotherapy (8, 19). In addition to inducing immunogenic cell death and antigen release, ADCs can also promote immune activation via Fc-mediated engagement of innate immune cells, dendritic cell activation, and increased neoantigen generation, thereby synergistically enhancing immune activation and tumor elimination (17, 20).

## 3 Clinical landscape and safety profile of ICI therapy in TNBC

### 3.1 Current clinical applications of ICIs in TNBC

**Figure 1:**
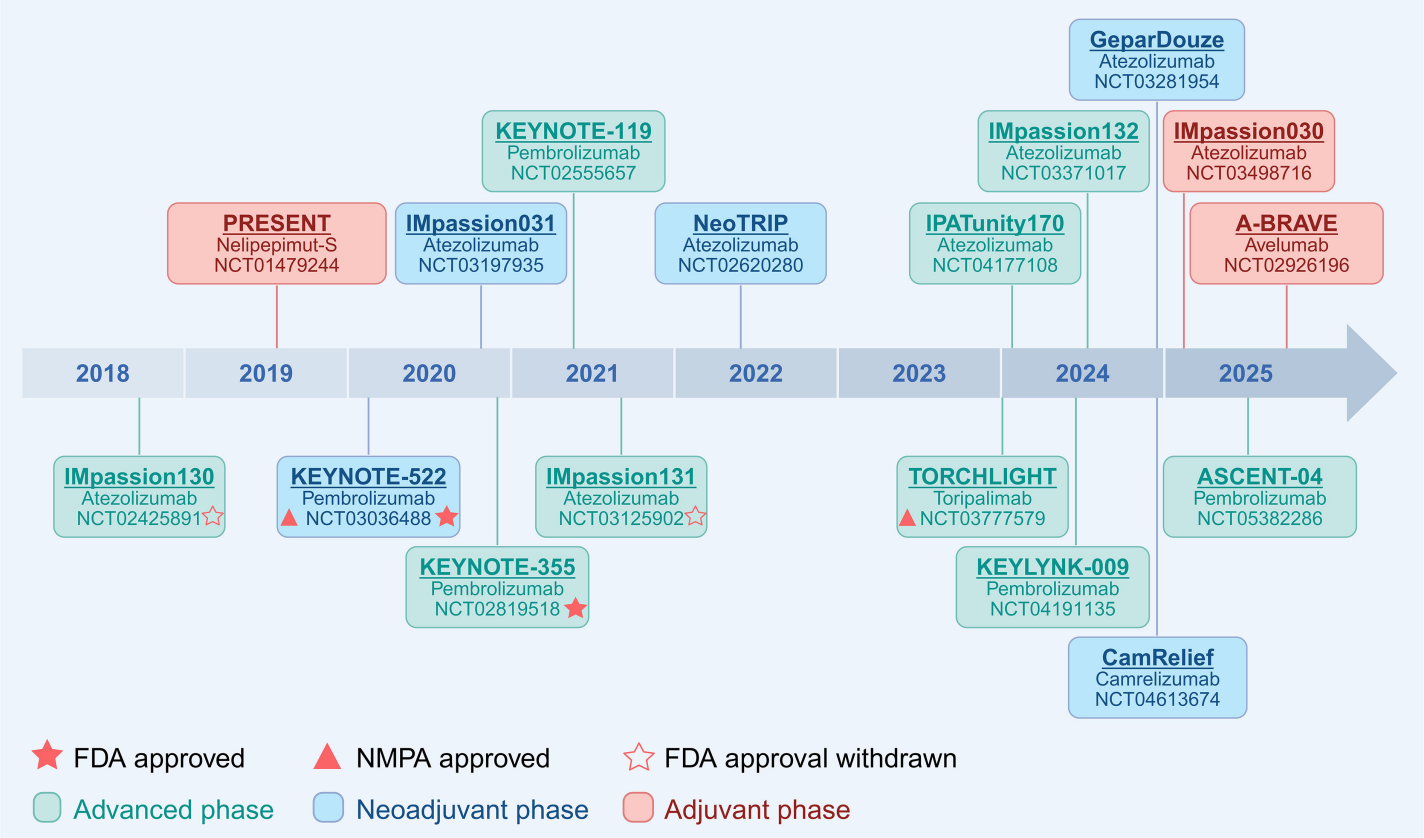
Timeline of phase III trials evaluating immunotherapy-based strategies in triple-negative breast cancer (TNBC). Clinical trials are arranged chronologically according to the publication date of their primary results. Each study is represented by a colored box corresponding to the treatment setting (neoadjuvant, adjuvant, or advanced). A red star (★) denotes U.S. Food and Drug Administration (FDA) approval, a hollow red star (☆) indicates an FDA-approved indication that was subsequently withdrawn, and a red triangle (▴) indicates approval by the National Medical Products Administration (NMPA). Created in BioRender. Zhou, Y. (2026) https://BioRender.com/e3ftf4i.

TNBC exhibits distinctive immunological traits, including elevated immune cell infiltration, upregulated PD−L1 expression, substantial genomic instability, and a higher mutational burden relative to other subtypes (21–24), making it particularly susceptible to immunotherapeutic approaches. A key advance in TNBC immunotherapy has been the application of ICIs, particularly monoclonal antibodies targeting the PD-1/PD-L1 axis. Agents such as pembrolizumab (PD-1 inhibitor), toripalimab (PD-1 inhibitor), and atezolizumab (PD-L1 inhibitor) have demonstrated meaningful clinical activity across multiple TNBC trials. By blocking inhibitory signals that suppress T-cell activation, these therapies enhance antitumor immunity and have translated into improvements in pathological response and/or survival outcomes (25). **Figure 1** presents a timeline of phase III trials evaluating immunotherapy-based strategies in TNBC.

Based on the IMpassion130 trial (26), the FDA granted accelerated approval in March 2019 to atezolizumab combined with albumin-bound paclitaxel for unresectable locally advanced or metastatic PD-L1–positive TNBC (27); however, after the confirmatory IMpassion131 trial failed to meet its endpoints (28) and the final analysis of IMpassion130 showed no statistically significant OS benefit in the intention-to-treat population (29), Roche voluntarily withdrew this indication in 2021. In contrast, supported by the KEYNOTE-355 trial (30), pembrolizumab received accelerated approval in 2020 and subsequent regular approval in 2021 from the FDA for locally recurrent unresectable or metastatic TNBC with PD-L1 expression (Combined Positive Score [CPS] ≥10), with confirmatory data from the KEYNOTE-522 trial (5), conducted in an earlier disease setting in accordance with FDA guidance permitting confirmatory trials to be performed in earlier stages of the same tumor type (31). Meanwhile, the KEYNOTE-522 trial (5, 6) led to FDA and NMPA approval of pembrolizumab for high-risk early-stage TNBC in combination with NACT followed by adjuvant monotherapy, with NMPA approval requiring PD-L1 expression (CPS ≥20). In addition, based on the TORCHLIGHT study (32), toripalimab combined with albumin-bound paclitaxel was approved by the NMPA on June, 2024, for PD-L1–positive (CPS ≥1) recurrent or metastatic TNBC.

### 3.2 Common irAEs associated with ICI therapy in TNBC and their management

Although ICIs have significantly expanded the therapeutic landscape of TNBC, their irAEs have emerged as an increasingly important clinical concern. Unlike conventional chemotherapy-related toxicities, irAEs result from nonspecific immune activation and may affect almost any organ system. The most commonly involved organs include the skin, endocrine glands, gastrointestinal tract, liver, and lungs, although severe and occasionally life-threatening events can also occur in less commonly affected systems (16, 33, 34). In general, gastrointestinal toxicity and hypophysitis are more common with CTLA-4 inhibitors, whereas pulmonary toxicity and thyroid dysfunction are reported more frequently with anti-PD-1/PD-L1 agents (16). In early-stage TNBC, the KEYNOTE-522 trial further highlighted the clinical relevance of this issue, showing that pembrolizumab combined with chemotherapy was associated with a meaningful burden of immune-mediated toxicity, with thyroid disorders and severe skin reactions among the more common events and a small proportion of patients experiencing high-grade or even fatal irAEs (7). Collectively, these findings indicate that the clinical benefit of ICIs in TNBC should be interpreted together with careful attention to toxicity surveillance and supportive management.

The management of irAEs relies primarily on early recognition and timely intervention. Low-grade irAEs can often be managed with continued ICI treatment and close monitoring, whereas higher-grade toxicities may require treatment interruption and systemic corticosteroids, with additional immunosuppressive therapy reserved for refractory cases (35). Prophylactic corticosteroids are not routinely recommended, as there is currently no clinical evidence supporting pharmacologic prevention of irAEs (34, 36), and unnecessary immunosuppression could potentially attenuate antitumor activity (37–39). In clinical practice, risk mitigation relies on baseline assessment, patient education, regular laboratory monitoring, prompt evaluation of new symptoms, and early multidisciplinary collaboration (33, 40, 41). Although host factors such as pre-existing autoimmune disease, genotype, and the gut microbiome may influence susceptibility to irAEs, clinically validated predictive biomarkers are not yet available for routine use (33, 34, 42). Ongoing studies are exploring more precise immunomodulatory approaches, including agents targeting specific inflammatory pathways and biomarker-guided individualized strategies, to reduce irAEs while preserving antitumor efficacy (37–39, 42).

## 4 Current evidence on ICI therapy in early-stage TNBC

### 4.1 Evidence from neoadjuvant trials

The encouraging results of the KEYNOTE-522 trial have been paralleled by investigations into other PD-1/PD-L1 inhibitors in the neoadjuvant setting, albeit to varying degrees (**Table 1**). Phase III trials such as GeparDouze (atezolizumab; paclitaxel plus carboplatin followed by epirubicin/doxorubicin plus cyclophosphamide) (43), IMpassion031 (atezolizumab; nab-paclitaxel followed by doxorubicin plus cyclophosphamide) (44, 45), and CamRelief (camrelizumab; nab-paclitaxel plus carboplatin followed by epirubicin plus cyclophosphamide) (46) have demonstrated encouraging activity, showing notable improvements in pCR rates when ICIs were added to NACT. However, not all neoadjuvant studies have produced positive outcomes. The phase III NeoTRIP trial, which also evaluated atezolizumab in combination with chemotherapy, failed to demonstrate significant improvements in either pCR (47) or survival (48). Differences in trial design, such as the omission of anthracycline-based regimens during the neoadjuvant setting in NeoTRIP, may have contributed to these discrepant results. Beyond phase III evidence, a recently published Perspective article reviewed the available phase II neoadjuvant trials of ICIs in TNBC, noting similarly heterogeneous findings but a possible tendency toward improved efficacy when ICIs were combined with chemotherapy (17). Notably, the phase II GeparNuevo trial provided intriguing insights: although the addition of durvalumab to standard NACT yielded only a modest increase in pCR (49), it translated into meaningful long-term survival benefits, even without adjuvant durvalumab administration (50).

**Table 1:** Phase III immune checkpoint inhibitor (ICI)-based trials with reported outcomes in early-stage triple-negative breast cancer (TNBC).

Trial	Population	Sample size	Neoadjuvant regimen	pCR rate	Adjuvant regimen	Survival outcomes	Safety	Reference
Neoadjuvant setting								
NeoTRIP (NCT02620280)	Early-stage TNBC	280 (138, 142)	Arm A: atezolizumab + nab-PCb; Arm B: nab-PCb	48.6% vs. 44.4% (*P* = 0.48)	Anthracycline regimen in both arms	5-year EFS: 70.6% vs. 74.9% (*P* = 0.76)	Grade ≥3 TRAEs: 77.5% vs. 70%	pCR (47), 5-year survival (48)
Neoadjuvant and adjuvant setting								
KEYNOTE-522 (NCT03036488)	Stage II-III TNBC	1174 (784, 390)	Arm A: pembrolizumab + PCb → pembrolizumab + AC/EC; Arm B: placebo + PCb → placebo + AC/EC	64.8% vs. 51.2% (***P* < 0.001**)	Arm A: pembrolizumab; Arm B: placebo	5-year EFS: 81.2% vs. 72.2% (***P* < 0.001**); 5-year OS: 86.6% vs. 81.7% (***P* = 0.002**)	Grade ≥3 TRAEs: 77.1% vs. 73.3%	pCR (5), 3-year survival (6), 5-year survival (7), RCB analysis (51)
IMpassion031 (NCT03197935)	Stage II-III TNBC	333 (165, 168)	Arm A: atezolizumab + wnab-P → atezolizumab + ddAC; Arm B: placebo + wnab-P → placebo + ddAC	58% vs. 41% (***P* = 0.0044**)	Arm A: atezolizumab + SoC; Arm B: SoC	2-year EFS and OS were not significantly improved	Grade ≥3 TRAEs: 56.7% vs. 53.3%	pCR (44), 2-year survival (45)
GeparDouze (NCT03281954)	Stage II-III TNBC	1550 (773, 777)	Arm A: atezolizumab + wPCb → atezolizumab + AC/EC; Arm B: placebo + wPCb → placebo + AC/EC	63.3% vs. 57.0% (***P* = 0.009**)	Arm A: atezolizumab; Arm B: placebo	4-year EFS: 85.2% vs. 81.9% (*P* = 0.08); 4-year OS: 90.2% vs. 89.5%	Grade ≥3 TEAEs: 75.3% vs. 73.4%	(43)
CamRelief (NCT04613674)	Stage II-III TNBC	441 (222, 219)	Arm A: camrelizumab + nab-PCb → camrelizumab + EC; Arm B: placebo + nab-PCb → placebo + EC	56.8% vs. 44.7% (***P* = 0.004**)	Arm A: camrelizumab + SoC; Arm B: SoC	NR	Grade ≥3 AEs: 89.2% vs. 83.1%	(46)
Adjuvant setting								
IMpassion030 (NCT03498716)	Postoperative stage II-III TNBC	2199 (1011, 1098)	/	/	Arm A: atezolizumab + wP → atezolizumab + ddAC/EC → atezolizumab; Arm B: wP → ddAC/EC	3-year iDFS and OS were not improved	Grade ≥3 TRAEs: 54% vs. 44%	(52)
A-BRAVE (NCT02926196)	Stage IIb-III or non-pCR, TNBC or HR-low/HER2-	466 (235, 231)	SoC	/	Arm A: curative-intent therapy → avelumab; Arm B: curative-intent therapy → observation	3-year DFS: 68.3% vs. 63.2% (*P* = 0.172); 3-year OS: 84.8% vs. 76.3% (**hazard ratio 0.66, 95% CI 0.45-0.97**)	Grade ≥3 irAEs were rare in both arms	(8)

pCR, pathological complete response; PCb, paclitaxel and carboplatin; EFS, event-free survival; AC, doxorubicin and cyclophosphamide; EC, epirubicin and cyclophosphamide; OS, overall survival; TRAEs, treatment-related adverse events; wnab-P: weekly nanoparticle albumin-bound paclitaxel; dd, dose-dense; SoC, standard of care; wP, weekly paclitaxel; TEAEs, treatment-emergent adverse events; NR, not reported. AEs, adverse events; iDFS, invasive disease-free survival; DFS, disease-free survival; HR-low, hormone receptor–low; HER2, human epidermal growth factor receptor 2; CI, confidence interval; irAE, immune-related adverse event. Bold indicates statistical significance.

### 4.2 Evidence from adjuvant trials

To date, only two phase III trials have reported data on the addition of ICIs to adjuvant chemotherapy in early-stage TNBC. Among them, the IMpassion030 trial was the first phase III study to evaluate adjuvant atezolizumab combined with chemotherapy in this population, with the primary endpoint being invasive DFS. In this trial, 2199 patients with resected early-stage TNBC who had not received any prior systemic therapy were randomly assigned (1:1) to receive atezolizumab plus standard adjuvant chemotherapy or adjuvant chemotherapy alone. However, its final analysis revealed that the addition of atezolizumab did not significantly improve invasive DFS or OS (52). Another phase III trial, A-BRAVE, compared one year of adjuvant avelumab versus observation in patients with early-stage TNBC who either had residual disease after NACT (383/466, 82.2%) or were considered at high risk following primary surgery and adjuvant chemotherapy (83/466, 17.8%). Although adjuvant avelumab did not significantly improve DFS (primary endpoint), descriptive and exploratory analyses suggested that avelumab conferred a benefit in 3-year OS (secondary endpoint) and distant DFS (8). This unexpected observation warrants validation in larger cohorts before its potential clinical relevance can be established.

### 4.3 Mechanistic insights into optimal timing of ICI use

**Figure 2:**
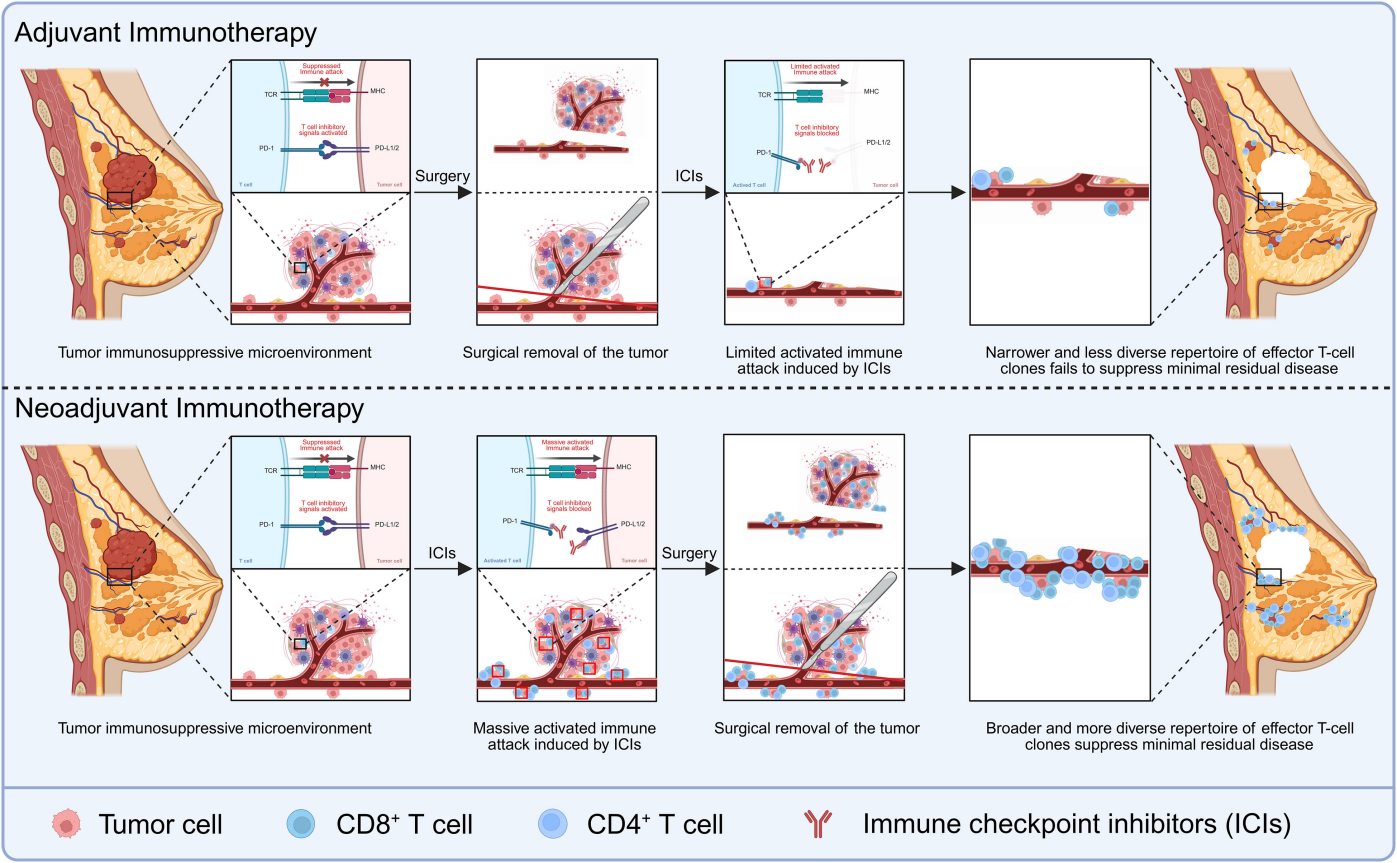
Immunological rationale for neoadjuvant versus adjuvant immunotherapy. The lower panel depicts the neoadjuvant setting, in which immune checkpoint inhibitors (ICIs) are administered before surgery while the primary tumor is still present. The presence of abundant tumor antigens facilitates more effective immune activation and promotes the development of a broader and more diverse repertoire of effector T-cell responses, leading to enhanced antitumor immunity. In contrast, the upper panel illustrates the adjuvant setting, where ICIs are given after surgery. Following tumor removal, antigen availability is reduced, resulting in relatively limited activation of antitumor T-cell responses and less effective immune control of minimal residual disease. Created in BioRender. Zhou, Y. (2026) https://BioRender.com/iv7shsm.

From a mechanistic perspective, neoadjuvant initiation of immunotherapy confers a distinct biological advantage over adjuvant use alone (52). Unlike cytotoxic chemotherapy that directly eliminates tumor cells, ICI restore antitumor immunity by reversing tumor-induced immune suppression. **Figure 2** illustrates the immunological rationale for neoadjuvant versus adjuvant immunotherapy. The neoadjuvant setting provides an ideal window for immune activation, as the TME remains immunologically active and antigen exposure is abundant, allowing ICIs to induce stronger and more durable antitumor responses when macroscopic tumor antigens are present (18, 53–55). Consistently, both preclinical models and studies in other tumor types have demonstrated enhanced ICI efficacy when administered in the neoadjuvant setting (56, 57). Administering ICIs in the presence of the intact tumor can therefore induce a broader and more diverse repertoire of effector T-cell clones, potentially establishing long-term immune memory that suppresses minimal residual disease after surgery. In contrast, when ICIs are initiated only in the adjuvant setting after complete tumor resection, the absence of a tumor antigen reservoir may limit immune priming and reduce the magnitude of the antitumor response (52).

## 5 Probing future directions for ICI-based therapy in early-stage TNBC

### 5.1 Optimizing the timing of ICI therapy

Although both mechanistic reasoning and emerging clinical data suggest stronger immunogenicity and clinical benefit when ICIs are delivered preoperatively, as discussed above, it remains important to consider whether postoperative pembrolizumab may still offer benefit for certain patient subgroups. A clinically relevant question arises in patients who did not receive ICIs during the neoadjuvant phase, particularly those who fail to achieve pCR and thus carry a substantially higher risk of recurrence. For such individuals, the potential role of adjuvant ICI as a “salvage” or compensatory strategy warrants careful consideration.

As noted earlier, A-BRAVE enrolled predominantly high-risk patients who had residual disease after NACT. Although adjuvant avelumab did not improve DFS, it yet indicated a possible benefit in OS, suggesting that some non-pCR patients may still derive value from adjuvant immunotherapy (8). Further clarity is expected from the ongoing phase III SWOG S1418/KEYNOTE-242 trial (NCT02954874), which is evaluating adjuvant pembrolizumab versus observation in TNBC patients with ≥1 cm residual invasive disease or any nodal involvement after completion of NACT and surgery (**Table 2**). Given that this trial enrolls patients with more extensive residual disease than the A-BRAVE trial and includes a larger population (>1000 patients), while using the same agent (pembrolizumab) that demonstrated robust efficacy in the KEYNOTE-522 trial, its results may show improved efficacy compared with the A-BRAVE trial and are thus eagerly awaited. Positive findings could help define whether adjuvant ICI provides incremental benefit in non-pCR patients following NACT.

**Table 2:** Ongoing phase III trials of immune checkpoint inhibitor (ICI)-based therapies in early-stage triple-negative breast cancer (TNBC).

Trial	Study completion (Est.)	Population	Size (Est.)	Regimen	Primary endpoint	Secondary endpoint
Neoadjuvant ± adjuvant setting						
NCT04907344	2025	Stage II-III TNBC	380	Arm A: camrelizumab + nab-PCb → surgery → SoC Arm B: nab-PCb → surgery → SoC	pCR	bpCR, ORR, EFS, iDFS, OS, safety
BCTOP-T-N01 (NCT05999149)	2027	Stage II-III TNBC	424	Arm A: camrelizumab + nab-PCb + famitinib → surgery → camrelizumab + famitinib; Arm B: camrelizumab + nab-PCb → surgery → camrelizumab	pCR	EFS, DFS, DDFS, ORR
ADAPT-TN-III (NCT06081244)	2029	Stage I or selected stage II TNBC or HR-low/HER2–	348	Arm A: pembrolizumab + SG ± NACT → surgery → ± TPC; Arm B: SG ± NACT → surgery → ± TPC	pCR, iDFS	OS, DDFS, DDFI, RFS, HRQoL
NCT06627712	2031	Early-stage (cT1cN1–2 or cT2N0-2) TNBC	120	Arm A: PD-1 inhibitor + SBRT + nab-PCb → PD-1 inhibitor + SBRT + EC → surgery → SoC Arm B: PD-1 inhibitor + nab-PCb → PD-1 inhibitor + EC → surgery → SoC	pCR	BCS, OS, safety, LRR
TROPION-Breast04 (NCT06112379)	2032	Stage II-III TNBC or HR-low/HER2–	1902	Arm A: durvalumab + Dato-DXd → surgery → durvalumab ± chemotherapy ± olaparib; Arm B: KEYNOTE-522 regimen → surgery → pembrolizumab ± capecitabine ± olaparib	EFS	pCR, OS, DDFS, PROs, PK, immunogenicity, safety
SWOG 2212 (NCT05929768)	2033	Early-stage (cT2-4N0 or cT1-3N1-2) TNBC or HR-low/HER2–	2400	Arm A: docetaxel + carboplatin + pembrolizumab → surgery → ± pembrolizumab; Arm B: KEYNOTE-522 regimen → surgery → ± pembrolizumab	BC-EFS	pCR, RCB, DRFS, OS, RFS, safety, PROs
ADAPT-TN-IV (NCT07178730)	2033	Stage II-III TNBC or HR-low/HER2–	765	All: pembrolizumab + PCb + evaluation Cohort 1 (Stage II and cCR): → surgery → SoC; Cohort 2 (Stage III or non-cCR): → Arm A: pembrolizumab + SG → surgery → SoC; Arm B: pembrolizumab +AC/EC → surgery → SoC	EFS, pCR	Clinical response, DDFS, RFS, OS, HRQoL
TroFuse-032 (NCT06966700)	2034	Early-stage (cT1cN1–2 or cT2-4N0-2) TNBC or HR-low/HER2–	2400	Arm A: pembrolizumab + sac-TMT → pembrolizumab + wPCb → surgery → pembrolizumab ± capecitabine ± olaparib ± AC/EC; Arm B: KEYNOTE-522 regimen → surgery → pembrolizumab ± capecitabine ± olaparib	pCR, EFS	OS, HRQoL, safety
Adjuvant setting						
SWOG S1418 (NCT02954874)	2026	Non-pCR (ypT ≥1 cm and/or ypN1mi–N3) TNBC or HR-low/HER2–/equivocal after NACT (without ICI)	1155	Arm A: pembrolizumab; Arm B: observation	iDFS, PROs	OS, DRFS, safety, HRQoL, biomarkers
TROPION-Breast03 (NCT05629585)	2030	Non-pCR TNBC after neoadjuvant therapy	1174	Arm A: durvalumab + Dato-DXd; Arm B: Dato-DXd; Arm C: capecitabine ± pembrolizumab	iDFS	OS, DDFS, PROs, PK, immunogenicity, safety, tolerability
NCT06533384	2030	Non-pCR TNBC after neoadjuvant therapy	310	Arm A: camrelizumab + fuzuloparib (gBRCA1/2-mut) or capecitabine (gBRCA1/2-wt); Arm B: camrelizumab	iDFS	OS, DDFS, safety
ASCENT-05 (NCT05633654)	2031	Non-pCR TNBC or HR-low/HER2– after neoadjuvant therapy	1514	Arm A: pembrolizumab + SG; Arm B: pembrolizumab ± capecitabine	iDFS	OS, DDFS, RFS, safety, HRQoL
OptimICE-pCR (NCT05812807)	2033	pCR TNBC or HR-low/HER2– after NACT plus pembrolizumab	1295	Arm A: observation; Arm B: pembrolizumab	RFS	Safety, OS, LRR
NCT06313463	2035	Non-pCR TNBC after neoadjuvant NACT plus camrelizumab	375	Arm A: camrelizumab + capecitabine; Arm B: placebo + capecitabine	DFS	iDFS, OS, DRFI, PROs
TroFuse-012 (NCT06393374)	2037	Non-pCR TNBC after neoadjuvant KEYNOTE-522 regimen	1530	Arm A: pembrolizumab + sac-TMT; Arm B: pembrolizumab ± capecitabine	iDFS	OS, DRFS, HRQoL, safety
OPT-PEMBRO (NCT06606730)	2039	pCR TNBC or HR-low/HER2– after NACT plus pembrolizumab	2454	Arm A: observation; Arm B: pembrolizumab	RFS	Safety, PROs, HRQoL, IBCFS, DRFS, OS

Est., estimated; nab-PCb, nanoparticle albumin-bound paclitaxel and carboplatin; SoC, standard of care; pCR, pathological complete response; bpCR, breast pathological complete response; ORR, objective response rate; EFS, event-free survival; iDFS, invasive disease-free survival; OS, overall survival; DDFS, distant disease-free survival; HR, hormone receptor; HER2, human epidermal growth factor receptor 2; SG, sacituzumab govitecan; NACT, neoadjuvant chemotherapy; TPC, treatment of physician’s choice; DDFI, distant disease-free interval; RFS, relapse-free survival; HRQoL, health-related quality of life; PD-1, programmed cell death protein 1; SBRT, stereotactic body radiotherapy; EC, epirubicin and cyclophosphamide; BCS, breast conservation rate; LRR, locoregional recurrence; Dato-DXd, datopotamab deruxtecan; KEYNOTE-522 regimen, pembrolizumab with carboplatin/taxanes followed by pembrolizumab with anthracycline/cyclophosphamide; PROs, patient-reported outcomes; PK, pharmacokinetics; BC-EFS, breast cancer event-free survival; RCB, residual cancer burden; DRFS, distant relapse-free survival; cCR, clinical complete response; AC, doxorubicin and cyclophosphamide; sac-TMT, sacituzumab tirumotecan; wP, weekly paclitaxel; gBRCA1/2, germline breast cancer susceptibility genes 1 and 2; mut, mutation; wt, wild type; DRFI, distant recurrence-free interval; IBCFS, invasive breast cancer–free survival.

Beyond treatment sequencing within the perioperative setting, the time of day (ToD) at which ICIs are administered has also attracted increasing interest. Accumulating evidence suggests that host circadian rhythms may influence immune activation, drug metabolism, and tumor progression (58). In cancers such as melanoma, renal cell carcinoma, and lung cancer, later ToD ICI infusions were associated with inferior survival outcomes (59, 60) In contrast, TNBC-specific data have not demonstrated a clinically relevant chronotherapeutic effect. In a single-center retrospective cohort of patients receiving the KEYNOTE-522 regimen, neither pembrolizumab nor chemotherapy infusion time was associated with pCR (58). Similarly, in the SIMCLOCK study of dose-dense NACT, morning versus afternoon infusion time did not affect pCR, residual cancer burden (RCB), metabolic response, toxicity, or short-term event-free survival (EFS) in early TNBC (61). More recently, PEMCLOCK, the first large real-world multicenter study investigating immunochemotherapy ToD in high-risk early-stage TNBC, reported preliminary data showing a numerical difference in pCR between the late (74%) and early (63%) ToD groups, while analyses of toxicity and survival outcomes remain ongoing (62). Taken together, the ToD of immunochemotherapy administration has not yet been shown to definitively affect response, toxicity, and survival in TNBC, and further confirmation from mature long-term follow-up and higher-level prospective studies is warranted.

### 5.2 Optimizing the duration of ICI therapy

In the adjuvant phase of KEYNOTE-522, grade ≥3 TRAEs occurred in 6.3% of patients (6), underscoring the potential risks and quality-of-life implications associated with continuing pembrolizumab after surgery. Notably, among patients who achieved pCR in KEYNOTE-522, the 3-year EFS was comparable between those receiving pembrolizumab plus chemotherapy and those receiving placebo plus chemotherapy (94.4% vs. 92.5%) (6). In contrast, in the GeparNUEVO trial, where no adjuvant ICI was administered, the durvalumab-chemotherapy group still demonstrated a superior 3-year iDFS compared with the placebo-chemotherapy group (95.5% vs. 86.1%) (50). Correspondingly, the necessity of continuing pembrolizumab postoperatively as mandated the in KEYNOTE-522 protocol, particularly for patients who achieve pCR, has become an increasingly debated question. Two ongoing international phase III trials, namely the OptimICE-pCR trial (NCT05812807) (63) and OPT-PEMBRO (NCT06606730), will address this issue. In these studies, an estimated 1295 and 2454 patients, respectively, with pCR after NACT plus pembrolizumab are randomized to observation versus continued adjuvant pembrolizumab to determine whether recurrence-free survival with observation is non-inferior to pembrolizumab monotherapy. The results of these two trials will evaluate whether adjuvant pembrolizumab can be safely omitted in the pCR cohort, aiming to support treatment de-escalation in this favorable-risk population.

For patients without pCR, continuation of pembrolizumab in the adjuvant setting is generally regarded as standard practice. In KEYNOTE-522, EFS outcomes varied across RCB categories, with hazard ratios (95% confidence intervals) of 0.70 (0.38–1.31) for RCB-0, 0.92 (0.39–2.20) for RCB-I, 0.52 (0.32–0.82) for RCB-II, and 1.24 (0.69–2.23) for RCB-III (51). These data indicate that the most pronounced relative advantage occurred in the RCB-II subgroup, suggesting that patients with RCB-II residual disease may be particularly positioned to benefit from continued adjuvant ICI therapy. Nevertheless, it is also possible that the observed survival benefit primarily reflects durable immune priming induced during the neoadjuvant phase, rather than the adjuvant therapy itself.

### 5.3 Assessing the potential of ICI dose de-escalation

In addition to treatment timing and duration, the feasibility and potential of reduced-dose pembrolizumab in early-stage TNBC has recently received attention. Pharmacodynamic studies of PD-1 inhibitors have consistently shown near-complete receptor saturation at doses substantially lower than those currently adopted in clinical practice (64–66), providing a biological rationale for dose de-escalation without necessarily compromising antitumor efficacy. Consistent with this concept, low-dose immune checkpoint inhibition has demonstrated clinical activity across multiple malignancies (67–71), supporting the feasibility of alternative dosing strategies.

The phase II PLANeT trial represents the first randomized study to directly explore a low-dose pembrolizumab approach in the neoadjuvant treatment of early-stage TNBC (72). In this single-center trial, the addition of pembrolizumab administered at 50 mg every 6 weeks for a total of three cycles (less than one-tenth of that of the standard pembrolizumab schedule) to standard neoadjuvant chemotherapy significantly increased the pCR rate compared with NACT alone (53.8% vs. 40.5%, respectively), with an absolute improvement comparable to that observed in KEYNOTE-522 (5), while not increasing the incidence of grade ≥3 toxicities. Although limited by its sample size and single-center design, PLANeT highlights the potential of dose de-escalation strategies to preserve efficacy while improving treatment accessibility and cost-effectiveness. These findings warrant further validation in larger, multicenter trials to confirm efficacy, safety, and feasibility of low-dose pembrolizumab within the KEYNOTE-522 framework.

### 5.4 Exploring novel ICI-based combination strategies

Despite its remarkable efficacy, the KEYNOTE-522 regimen carries substantial toxicity, with grade ≥3 treatment-related adverse events (TRAE) in 77.1% of patients and 4 deaths (0.5%), most of which occurred during the neoadjuvant phase (7). These potentially life-threatening and long-lasting toxicities highlight the need to optimize the chemotherapy backbone and explore treatment de-escalation to balance efficacy and safety. An important future direction is whether the current NACT backbone can be safely de-escalated, replaced, or even omitted in selected TNBC patients without compromising efficacy.

Emerging evidence from trials such as NeoPACT (73), NCI 10013 (with adjuvant doxorubicin) (74), and cTRIO (75) indicates that anthracycline-free regimens, when combined with ICIs, can achieve compelling pCR rates and encouraging early survival outcomes. These findings challenge the indispensable role of anthracyclines in this setting and highlight a growing interest in treatment de-escalation to mitigate toxicity. The ongoing phase III SWOG 2212/SCARLET trial (NCT05929768) is expected to provide pivotal evidence by directly comparing an anthracycline-free immunochemotherapy regimen with the established KEYNOTE-522 protocol, potentially redefining the future SoC. In addition, carboplatin-free regimens have also attracted attention. Single-arm phase II studies such as NCT04213898 (76), TREND (77), and NeoTENNIS (78) have demonstrated encouraging pCR rates of 25/39 (64.1%), 30/44 (68.2%), and 39/70 (55.7%), respectively. Furthermore, in the two-arm trials IMpassion031 (44, 45) and GeparNuevo (49, 50), which compared carboplatin-free chemotherapy with or without ICIs, the addition of ICI produced paradoxical results with respect to pathological response and survival outcomes. At present, the available data are insufficient to support the de-escalation of chemotherapy backbone from the KEYNOTE-522 regimen, although treatment decisions may reasonably be individualized based on patient-specific toxicity profiles.

Beyond immunochemotherapy, novel ICI-based combinations, particularly ADCs combined with ICIs, have attracted increasing interest, as preclinical studies suggest synergistic antitumor activity (79, 80). Among ADCs, trophoblast cell surface antigen 2 (TROP2)–directed agents have already progressed from bench to clinical evaluation, with emerging clinical data supporting their therapeutic potential when combined with ICIs. In the phase Ib/II BEGONIA trial, the combination of datopotamab deruxtecan (Dato-DXd) and durvalumab demonstrated robust antitumor activity and a manageable safety profile as first-line therapy for advanced TNBC, achieving a confirmed objective response rate of 79% and a median progression-free survival (PFS) of 13.8 months at a median follow-up of 11.7 months (81). Notably, the phase III ASCENT-04/KEYNOTE-D19 trial showed that sacituzumab govitecan combined with pembrolizumab significantly improved PFS compared with chemotherapy plus pembrolizumab in previously untreated, PD-L1–positive advanced TNBC, while also exhibiting better tolerability, suggesting potential advantages beyond conventional immunochemotherapy (82). In the neoadjuvant setting, results from I-SPY 2.2 indicated modelled pCR rates of 43% in TNBC patients treated with only four cycles of Dato-DXd plus durvalumab, with immune-positive subtypes appearing to derive greater benefit (83). Although no additional clinical data have been disclosed to date, TROP2-directed ADCs combined with ICIs are considered to have the potential to challenge the current SoC defined by the KEYNOTE-522 regimen. Studies evaluating the neoadjuvant potential of ADC–ICI combinations in TNBC, including TROPION-Breast04, TroFuse-032, NeoSTAR (84), ADAPT-TN-III, and ADAPT-TN-IV, have been comprehensively summarized in previous literature (17) and are therefore not reiterated here.

Checkpoint-targeting bispecific antibodies represent another emerging ICI-based strategy. By simultaneously targeting two distinct tumor-promoting pathways, these agents may enhance antitumor activity and help overcome resistance (85). In the first-line treatment of metastatic TNBC, ivonescimab, a PD-1/VEGF-A bispecific antibody, has shown encouraging activity with an ORR of 72.4% (21/29) when combined with chemotherapy (86), while KN046, a PD-L1/CTLA-4 bispecific antibody, has also demonstrated promising activity with tolerable toxicity (87). More importantly, in the neoadjuvant setting, the phase II CABIN study showed that cadonilimab (AK104), a PD-1/CTLA-4 bispecific antibody, combined with nab-paclitaxel and carboplatin achieved a total pCR rate of 65.5% (19/29) and an ORR of 93.1% (27/29), with a manageable safety profile (88), suggesting promising activity in early-stage TNBC. In addition, a phase II study of ivonescimab combined with NACT is ongoing (NCT06977542).

Other exploratory strategies have also been explored. Dual immune checkpoint blockade, exemplified by nivolumab (a PD-1 inhibitor) combined with ipilimumab (a CTLA-4 inhibitor), has shown promise in the neoadjuvant setting; however, substantial immune-related toxicity may restrict its clinical applicability (89, 90). Local immunomodulatory approaches, including radiotherapy (e.g (91) and the phase III NCT06627712 trial), cryoablation (e.g (92) and high-intensity focused ultrasound (e.g., NCT05491694), are also being actively investigated in combination with neoadjuvant ICIs for TNBC. For instance, preliminary results suggest that neoadjuvant stereotactic body radiotherapy combined with adebrelimab and chemotherapy can achieve pCR rates up to 90%, with tolerable toxicity profiles, as grade 3 or higher adverse events occurred in 53.8% of patients (91). Notably, radiotherapy may exert a Janus-faced effect when combined with immunotherapy in early-stage TNBC, enhancing the antitumor immune response through increased antigen release, greater infiltration of tumor-infiltrating lymphocytes, and remodeling of the TME, while also increasing immune-related toxicity and paradoxically inducing immunosuppression through the recruitment of suppressive immune cells (93–96). Robust prospective evidence is still needed to optimize the dose, fractionation, and sequencing of radiotherapy in this setting (94, 95).

### 5.5 Refining patient stratification and treatment customization in early-stage TNBC

Despite the efficacy of standard neoadjuvant immunochemotherapy, a subset of patients with early-stage TNBC still derive limited benefit. This variability largely reflects the marked biological and immunological heterogeneity of TNBC. Tumors with an immune-cold or immunosuppressive microenvironment and limited baseline immune activation may derive less benefit from neoadjuvant immunochemotherapy (97–99). Age-related immunosenescence may also contribute to suboptimal antitumor immune responses in older patients (17). At the molecular level, TNBC can be further divided into multiple subtypes, and growing evidence suggests that responses to both immunotherapy (100, 101) and chemotherapy (102) may differ across these subgroups. For instance, the immunomodulatory subtype is generally considered more likely to benefit from immunotherapy (100), whereas the mesenchymal-like subtype has been associated with reduced sensitivity to ICIs (101). Future research should therefore aim to clarify the molecular and immune basis of response heterogeneity and resistance, thereby supporting the identification of predictive biomarkers and the rational design of future neoadjuvant trials.

Although PD-L1 expression, typically assessed by CPS, has been incorporated into regulatory approvals for immunotherapy in advanced TNBC and, in China, also in early-stage TNBC, its value as a predictive biomarker in early-stage disease remains less well defined (17, 25, 74). In the neoadjuvant setting, the predictive performance of PD-L1 has been inconsistent across studies (6, 44, 46, 47, 73, 74), and current evidence does not support treatment decisions being based solely on CPS (103). Tumor-infiltrating lymphocyte (TIL) levels appear promising as a predictive biomarker in early-stage TNBC, as patients with higher TIL levels are more likely to achieve pCR following neoadjuvant immunochemotherapy in both prospective trials (49, 73) and real-world cohorts (104). Other emerging biomarkers, including high tumor mutational burden (TMB-H), microsatellite instability-high (MSI-H)/mismatch repair deficient (dMMR) status, and immune-related gene expression signatures, may further refine prediction (25, 73, 105–109), although their clinical utility in early-stage TNBC remains limited by low prevalence, limited availability, and inconsistent validation across studies (17, 110–112). Overall, treatment customization in early-stage TNBC will likely require the integration of multiple clinicopathologic, immune, and molecular factors, including TILs, PD-L1, and other immunogenomic markers, to improve patient stratification and guide individualized neoadjuvant ICI-based strategies.

## 6 Conclusion

TNBC represents a highly immunogenic subtype amenable to ICIs. While the KEYNOTE-522 regimen has established the current standard of care by demonstrating remarkable efficacy, its substantial toxicity and the uncertainty regarding the respective contributions of neoadjuvant versus adjuvant administration underscore the need for further optimization. Consistent biological rationale and accumulating preclinical and clinical evidence indicate superior antitumor activity when ICIs are administered in the neoadjuvant setting, although adjuvant administration may also retain potential. The ongoing trials are exploring optimization strategies for the KEYNOTE-522 regimen, including treatment de-escalation and alternative immunotherapy partners, and may refine the timing, duration, dose, combination, and patient selection of ICIs in early-stage TNBC, potentially reshaping the therapeutic landscape and informing individualized treatment approaches.

## Glossary

AC: doxorubicin and cyclophosphamide
ADCs: antibody–drug conjugates
AEs: adverse events
BTEs: bispecific T-cell engagers
BC-EFS: breast cancer event-free survival
BCS: breast conservation rate
bpCR: breast pathological complete response
CAR: chimeric antigen receptor
CI: confidence interval
CPS: Combined Positive Score
CTLA-4: cytotoxic T-lymphocyte–associated protein 4
dd: dose-dense
DDFS: distant disease-free survival
DDFI: distant disease-free interval
DFS: disease-free survival
DRFI: distant recurrence-free interval
DRFS: distant relapse-free survival
EC: epirubicin and cyclophosphamide
EFS: event-free survival
FDA: U.S. Food and Drug Administration
gBRCA1/2: germline breast cancer susceptibility genes 1 and 2
HER2: human epidermal growth factor receptor 2
HR: hormone receptor
HR-low: hormone receptor–low
HRQoL: health-related quality of life
IBCFS: invasive breast cancer–free survival
ICIs: immune checkpoint inhibitors
iDFS: invasive disease-free survival
irAEs: immune-related adverse events
LRR: locoregional recurrence
mut: mutation
MSI-H/dMMR: microsatellite instability-high/mismatch repair deficient
NACT: neoadjuvant chemotherapy
nab-P: nanoparticle albumin-bound paclitaxel
NMPA: National Medical Products Administration
NR: not reported
ORR: objective response rate
OS: overall survival
PCb: paclitaxel and carboplatin
PD-1: programmed cell death protein 1
PD-L1: programmed death-ligand 1
PFS: progression-free survival
pCR: pathological complete response
PK: pharmacokinetics
PROs: patient-reported outcomes
RCB: residual cancer burden
RFS: relapse-free survival
SBRT: stereotactic body radiotherapy
SG: sacituzumab govitecan
SoC: standard of care
STING: stimulator of interferon genes
TEAEs: treatment-emergent adverse events
TIL: tumor-infiltrating lymphocyte
TMB-H: high tumor mutational burden
TME: tumor microenvironment
TNBC: triple-negative breast cancer
ToD: time of day
TRAE(s): treatment-related adverse event(s)
TPC: treatment of physician’s choice
wnab-P: weekly nanoparticle albumin-bound paclitaxel
wP: weekly paclitaxel
wt: wild type.
